# Maternal and infant outcomes of pregnancy anemia at one month postpartum: a cohort analysis

**DOI:** 10.1186/s12884-025-08137-3

**Published:** 2025-10-08

**Authors:** Lucienne Zinsstag, Souliviengkham Sonephet, Jessica Rigutto-Farebrother, Somphou Sayasone, Günther Fink, Jordyn T. Wallenborn

**Affiliations:** 1 Lao Tropical and Public Health Institute, Vientiane, Lao People’s Democratic Republic; 2 Department of Epidemiology and Public Health, Swiss Tropical and Public Health Institute, Allschwil, Switzerland; 3 University of Basel, Basel, Switzerland; 4 Institute of Food, Nutrition and Health, Department of Health Sciences and Technology, ETH Zürich, Zürich, Switzerland

**Keywords:** Anemia, Postpartum, Pregnancy, Mothers, Infant, Growth, Health, Anthropometrics, Vientiane capital, Lao PDR

## Abstract

**Background and objectives:**

Postpartum anemia is a significant public health concern affecting both maternal and infant health. This study aimed to investigate the relationship between maternal anemia during pregnancy, and infant anthropometric measurements at one month postpartum, as well as postpartum anemia in mothers and infants.

**Methods:**

Data from the Social Transfer for Exclusive Breastfeeding (STEB) randomized controlled trial in Vientiane, Lao People’s Democratic Republic (Lao PDR), were analyzed. Linear and logistic regression models were used to calculate adjusted estimates, including beta and odds ratios, and 95% confidence intervals (CI) for maternal hemoglobin (Hb) levels and anemic status during pregnancy and at one month postpartum. Primary outcomes were infant anthropometric measurements, with secondary outcomes including infant and maternal Hb levels and anemic status at one month postpartum.

**Results:**

At one month postpartum, we identified 298 mothers (mean age: 27 years) and their healthy term infants who were born at a healthy birth weight. No significant association was found between maternal anemic status (yes/no) and Hb levels during pregnancy and infant anthropometric measurements at one month postpartum. A positive association was observed between maternal and infant Hb levels at one month postpartum, with a 0.2 g/L increase in infant Hb for every 1 unit increase in maternal Hb (β = 0.23, 95% CI: 0.05, 0.40, *p* < 0.01).

**Conclusion:**

These findings highlight the importance of early postpartum intervention and continued nutritional care for both mothers and infants during the postpartum period, especially for mothers who experienced anemia during pregnancy. Further research is needed to explore the long-term effects of maternal postpartum health on infant development and growth.

**Supplementary Information:**

The online version contains supplementary material available at 10.1186/s12884-025-08137-3.

## Background

Anemia is associated with increased morbidity and mortality in women and children [1]. Anemia hinders oxygen transport, potentially leading to preterm birth and low birth weight in infants, which can have lasting repercussions on their health and development [2, 3]. Research indicates that infants born to mothers with anemia tend to have lower birth weights, smaller head circumferences, and reduced height, suggesting a relationship between maternal anemia and healthy term infant anthropometric measurements [4–6]. Additionally, infants born to anemic mothers are at higher risk of low hemoglobin (Hb) levels [7] and lower iron reserves up to 6 months of age, even when they are born at term and with a normal birth weight [7, 8].

It has become increasingly accepted that maternal postpartum anemia plays an important role in infant health and growth through its association with post-partum depression [9, 10], failure of lactation [11], and impaired maternal-child bonding [12]. However, research investigating the impacts of anemia on infant anthropometrics has been limited by their cross-sectional nature and/or by only investigating pregnancy anemia [13, 14]. Additionally, when investigating the relationship between maternal anemia on infant anthropometric measurements, it is important to control for exclusive breastfeeding — as exclusively breastfed infants show leaner growth and slower weight gain compared with formula-fed infants [15–18]. To our knowledge there are no studies that investigate the association between maternal anemia and infant anthropometric measurements focusing on term infants with a healthy weight of > 2500 kg and who are exclusively breastfed.

The World Health Organization (WHO) and Academy of Pediatrics explicitly state that exclusive breastfeeding is the ideal nutrition for infants and sufficiently supports optimal growth for the first 6 months of life [19, 20]. However, knowledge gaps exist regarding the sufficiency of breast milk alone (i.e. exclusive breastfeeding) for providing optimum iron and supporting child growth, especially among mothers with anemia [21]. Studies have reported that infants who are exclusively breastfed for six months without iron supplementation may become iron deficient [19]. — leading to reports that exclusively breastfed infants should receive iron supplementation from 4 months of age [20] and/or complementary foods be introduced before 6 months of age [22]. These studies underline the fact that the link between iron status of exclusively breastfed infants in relation to iron levels of breast milk and maternal anemia is still not completely understood and needs further investigation [19–22]. Considering the slower weight gain trajectories of exclusively breastfed compared with formula-fed infants [15–17] it is important to investigate the relationship between dietary indicators such as exclusive breastfeeding and infant anthropometric outcomes [23].

Anthropometric measurements like weight-for-age z-score (WAZ) serve as indicators of child health, and development. Metrics such as height-for-age HAZ and WAZ allow for the identification of stunted, wasted, and overweight children, and is also a signal for and malnutrition [24]^,^ [25]. While studies have investigated the role of anemia on infant weight, height, and head circumference [4, 5], further research is warranted to explore the association between maternal anemia and additional anthropometric measurements like skinfold thickness and mid-upper arm circumference.

Our study focuses on a unique population in Southeast East Asia that is subject to poverty and food insecurity [26]. The main research objective is to investigate the prolonged impacts of maternal anemia during pregnancy on postpartum anemia in mothers, as well as in their exclusively breastfed, healthy birthweight infants, and to examine its association with infant anthropometric outcomes at one month postpartum.

## Methods

### Study source

**Fig. 1:**
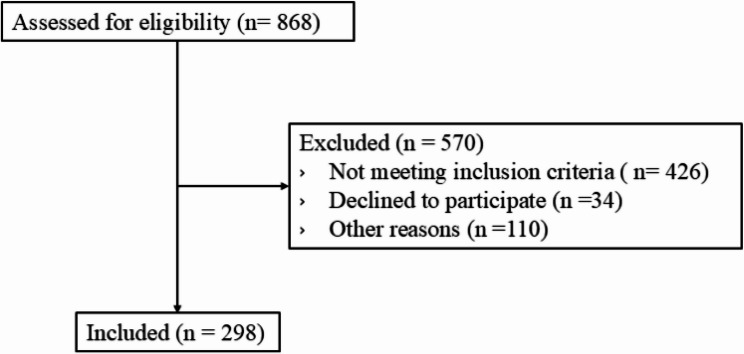
Flow of participant inclusion. A total of 868 women were identified through the Vientiane mulTi gEneRational Pre-BIrth cohort (VITERBI) and assessed for eligibility. Of these, 298 women were ultimately enrolled in the Social Transfers for Exclusive Breastfeeding (STEB) study

Our study analyzed data from the Social Transfers for Exclusive Breastfeeding (STEB) randomized control trial of 298 mothers and their infants in Vientiane, Lao People’s Democratic Republic (Lao PDR). Participants in STEB were first identified through the VIentiane mulTi gEneRational Pre-BIrth cohort (VITERBI) [27]. Women enrolled in VITERBI were called approximately one month after birth and enrolled in STEB [28] if they: (1) gave birth within the last four weeks, (2) were exclusively breastfeeding at time of recruitment, (3) had no illnesses that contraindicate breastfeeding, and (4) had a healthy singleton infant of 37 weeks or more gestation with a birth weight of at least 2500 g. The most common reason for exclusion was nonexclusive breastfeeding, defined as the provision of infant formula, water, or other supplements at the time of screening [29]. A detailed summary of the participant selection process is presented in Fig. 1 [29].

The STEB trial sample size was determined a priori to detect differences in exclusive breastfeeding outcomes, with a minimum required sample of 300 participants [28]. The present study constitutes a secondary analysis of the STEB dataset and was not independently powered to detect statistically significant differences in the specific maternal and infant outcomes assessed here.

### Study design

Our study strictly focuses on data from the VITERBI enrollment survey which takes place during the first, second and third trimesters of pregnancy and the baseline data collected prior to the implementation of the randomized intervention in the STEB trial. As such, the contents of the intervention are not relevant to the objectives or outcomes of the present study, which does not assess any post-intervention effects.

Women enrolled in VITERBI provided information on sociodemographic and lifestyle characteristics, including maternal smoking status, alcohol use, and survival history. The survival history data encompasses previous pregnancies and their outcomes. VITERBI serves as a basis for observational and interventional studies such as the STEB trial.

The STEB randomized control trial aimed to assess the effectiveness of social transfers; defined as a cash or in-kind transfer, on exclusive breastfeeding rates in Lao PDR. The baseline household visits were conducted between August and December 2022 in four districts of Vientiane, purposely selected based on their socioeconomic status: Chanthabuly and Sikhottabong as two urban districts with high socioeconomic status and Sangthong and Parkngum as rural districts with low socio-economic status. During the baseline visit, a questionnaire was administered, and bio specimens and anthropometric measurements were collected. The mothers answered questions about her work status, monthly income, and whether her newborn also received iron supplementation. Additionally, the mother was asked if her newborn had a doctor’s visit in the first month of life and if yes, what diagnosis the newborn received [28].

This study included questions from the VITERBI study such as marital status, the highest attained educational level, the amount of antenatal care visits, alcohol consumption during pregnancy, whether she received iron supplementation during pregnancy. Biospecimens included breastmilk (mothers), fecal samples (infant), Hb levels (mother and infant during and one month postpartum), and saliva (mother and infant). Anthropometric measurements of the infant and mother included weight, height, mid-upper arm circumference (MUAC), head circumference (HC), triceps- subscapular-, quadriceps-, and flank- skinfold thickness. The mothers’ anthropometric measurements were taken both during pregnancy and one month postpartum.

### Exposure variables

The primary exposure variables were maternal Hb levels and anemia status during pregnancy, which serve as a continuous and binary (anemia yes/no) variable. Following WHO guidelines from 2024, anemia during pregnancy was defined as Hb < 110 g/L for the first and third trimester and Hb < 105 g/L for the second trimester. The trimesters were calculated based on the child’s date of birth and the date of the first interview conducted in VITERBI. The secondary exposure variables were maternal Hb levels and anemia status at one month postpartum which was defined following WHO guidelines as Hb < 120 g/L [30]. Hb levels were assessed using HemoCue^®^ Hb 301 System from capillary blood samples collected at the time of the interview from both mother and infant. To standardize variations in iron levels over the day Hb measurements were taken at approximately the same time.

### Outcome variables

The primary outcome was measurements of infant anthropometry at one month postpartum, including infant length, referred here as height, weight, MUAC, HC, triceps-, subscapular-, quadriceps, and flank skinfold thickness. For all anthropometric measurements, the associated Z-scores for age were calculated using the World Health Organization (WHO) growth standards 2006 [31]. The WHO growth curves for MUAC, and the four different skinfold thicknesses only start at 3 months of age [31]; however, z-scores were calculated for all participants including those younger than 3 months, despite the lack of reference data for this younger age group. Infant height was measured in centimeters with the infant lying horizontal on a standardized infantometer. Weight was measured in kilograms using a mobile Scalter scale [32]. Skin fold measurements were collected using a skin fold caliper. HC and MUAC were measured using a non-stretchable measuring tape. A minimum of two measurements were taken for each planned measurement using standard techniques to reduce measurement bias. Measurements were repeated until two consecutive measurements were within 0.1 precision. The most common index for infant anthropometrics is birth weight for which the standard ranges 2500–4500 g [31]. Infant weight at one month postpartum is similar to birthweight due to loss and subsequent gain in infant weight during the first month postpartum [31]. Based on the WHO child growth standards the healthy range for infant height at birth is between 46.50 and 54.00 cm and for infant head circumference the standard lies between 32.0 and 37.0 cm [31].

Our secondary outcomes included postpartum Hb levels and the anemia status of both infants and mothers at one month postpartum. Anemia status was classified according to WHO hemoglobin thresholds from 2024 [29] and coded as a dichotomous variable (yes/no). Specifically, anemia was defined as Hb concentration < 105 g/L for infants and < 120 g/L for mothers during the postpartum period [30]. Due to the lack of WHO-recommended hemoglobin cutoffs specifically for one month old infants, we applied the threshold recommended for infants aged 6 months.

In this study, maternal Hb levels at one month postpartum were used both as an outcome—when examining the effect of pregnancy anemia—and as an exposure—when examining associations with infant outcomes. We acknowledge the potential concern of reverse causality in using maternal postpartum Hb in both roles; however, the analyses were structured to reflect plausible temporal relationships, and interpretations are made cautiously within the limitations of the study design.

### Confounders

A Directed Acyclic Graph (DAG) was developed to guide confounder selection based on theoretical considerations and prior literature and is presented in Supplementary Information Fig. 1. Based on this framework, we adjusted for the following confounding variables: infant biological sex (binary; female, male) and age (continuous) [33, 34], maternal BMI during pregnancy (categorical; underweight, normal, overweight) [35, 36], highest educational attainment (categorical; primary, secondary or university or higher), marital status (categorical; currently married, never married), antenatal care visits (ANC) (continuous) [37], urban or rural residence (binary; urban, rural), employment status (categorical; unemployed, employed, other), monthly income (continuous) [38], alcohol consumption (binary; yes, no) [35], and blood pressure both during pregnancy and one month postpartum (categorical; hypertension, normal range hypotension) [39, 40]. Categorizations of all confounders can be found in Tables 2 and 1 in the Appendix.

**Table 1:** Maternal descriptive statistics overall and by maternal anemic status (yes/no) during **a** pregnancy and **b** at one month postpartum

Characteristics		Study Population	Maternal anemic status pregnancy			Maternal anemic status one month postpartum		
			Non-Anemic	Anemic	*p*-value	Non-Anemic	Anemic	*p*-value
		*N* = 298	(*n* = 229)	(*n* = 65)		(*n* = 161)	(*n* = 134)	
(a) Pregnancy								
Area				0.865			0.861	
Urban	154 (51.7)	119 (52.0)	33 (50.8)		84 (51.2)	70 (52.2)		
Rural	144 (48.3)	110 (48.0)	32 (49.2)		80 (48.8)	64 (47.8)		
*Age (years)	27 (23, 31)	27 (27, 28)	27 (25, 28)	0.473	28 (27, 29)	26 (25, 28)	0.0673	
Trimester of Pregnancy				0.009			0.604	
1st trimester	27 (9.1)	22 (9.6)	5 (7.7)		16 (9.8)	11 (8.2)		
2nd trimester	107 (35.9)	93 (40.6)	14 (21.5)		62 (37.8)	45 (33.6)		
3rd trimester	164 (55.0)	114 (49.8)	46 (70.8)		86 (52.4)	78 (58.2)		
*Hemoglobin (g/L)	113.0 (105.0, 122.0)				116.3 (114.4, 118.2)	110.3 (108.0, 112.7)	0.000	
Anemic Status							0.018*	
Yes	65 (33.6)				79 (40.5)	55 (55.0)		
No	229 (65.4)				116 (59.5)	45 (45.0)		
Missing	4 (1.3)							
*Pre pregnancy Weight (Kg)	52.0 (47.0, 58.0)	58.5 (57.0, 60.0)	59.3 (56.8, 61.8)	0.634	53.6 (52.0, 55.3)	54.5 (52.3, 56.7)	0.519	
*Weight (Kg)	56.9 (50.6, 64.1)	54.1 (52.5, 55.6)	54.0 (51,4, 56.7)	0.978	58.5 (56.8, 60.2)	58.8 (56.9, 60.8)	0.813	
*Height (m)	1.5 (1.5, 1.6)	1.5 (1.5, 1.6)	1.5 (1.5, 1.5)	0.2452	1.5 (1.5, 1.6)	1.5 (1.5, 1.5)	0.5240	
Pre-pregnancy BMI (Kg/m^^2^)				0.637			0.004	
Underweight	35 (11.7)	28 (12.2)	7 (10.7)		12 (7.5)	23 (17.2)		
Normal	204 (68.5)	160 (69.9)	43 (66.2)		124 (77.0)	80 (59.7)		
Overweight	56 (18.8)	41 (17.9)	15 (23.1)		25 (15.5)	31 (23.1)		
Missing	3 (1.0)							
Blood Pressure (mm Hg)		(*n* = 126)	(*n* = 65)	0.701				
Hypertension								
Normal range		260 (87.3)	202 (89.4)	57 (87.7)				
Hypotension		32 (10.7)	24 (10.6)	8 (12.3)				
Missing		6 (2.0)						
*Income/per month (USD)	87.0 (17.4, 116.0)	178.3 (114.4, 242.3)	88.0 (65.6, 110.2)	0.141	145.0 (95.5, 194.5)	173.7 (80.4, 267.1)	0.5741	
Work Status				0.163			0.631	
Unemployed	94 (31.5)	65 (28.4)	20 (30.8)		46 (28.6)	48 (35.8)		
Employed	131 (44.0)	110 (48.0)	29 (44.6)		80 (49.7)	51 (38.1)		
Other	70 (23.5)	54 (23.6)	16 (24.6)		35 (21.7)	35 (26.1)		
Missing	3 (1.0)							
Education				0.313			0.320	
Primary	76 (25.5)	56 (24.5)	20 (30.7)		37 (23.0)	39 (29.1)		
Secondary	119 (39.9)	91 (39.7)	28 (43.1)		64 (39.8)	55 (41.0)		
University/Higher	100 (33.6)	82 (35.8)	17 (26.2)		60 (37.3)	40 (29.9)		
Missing	3 (1.0)							
Marital Status				0.536			0.738	
Never married	26 (8.7)	19 (8.3)	7 (10.8)		15 (9.3)	11 (8.2)		
Currently married	269 (90.3)	210 (91.7)	58 (89.2)		146 (90.7)	123 (91.8)		
Missing	3 (1.0)							
Alcohol consumption				0.227			0.529	
Yes	62 (20.8)	51 (22.3)	10 (15.4)		18 (11.2)	12 (9.0)		
No	233 (78.2)	178 (77.7)	55 (84.6)		143 (88.8)	122 (91.0)		
Missing	3 (1.0)							
Antenatal care visits	3.0 (2.0, 5.0)	3.4 (2.5, 4.2)	3.6 (3.3, 4.0)	0.7254	3.7 (2.5, 4.8)	3.2 (2.9, 3.5)	0.4981	
Iron Supplementation				0.092			0.604	
Yes	265 (88.9)	202 (88.2)	62 (95.4)		143 (88.8)	122 (91.0)		
No	30 (10.1)	27 (11.8)	3 (4.6)		18 (11.2)	12 (9.0)		
Missing	3 (1.0)							
(b) One Month Postpartum								
	*N* = 298	(*n* = 195)	(*n* = 100)		(*n* = 147)	(*n* = 128)		
*Hemoglobin level (g/L)	122.0 (113.0, 131.0)	122.7 (121.1, 124.3)	117.1 (114.1, 120.1)	0.001				
Anemic Status				0.003				
Yes	134 (45.0)	132 (57.6)	28 (43.1)					
No	164 (55.0)	97 (42.4)	37 (56.9)					
Blood Pressure (mm Hg)							0.748	
Hypertension		9 (3.0)				6 (3.7)	3 (2.2)	
Normal range		281 (94.3)				154 (93.9)	127 (94.8)	
Hypotension		8 (2.7)				4 (2.4)	4 (3.0)	

The categorical variables present total numbers and percentages. T-tests are presented as means and 95% confidence intervals (CI). Significance at *p* < 0.05. BMI is categorized as follows: Underweight (> 18.5); Normal weight (18.5 - <25) and Overweight (25.0 to 30+). Blood Pressure is categorized as follows: Hypertension = systolic blood pressure (SBP) ≥ 140 and diastolic blood pressure (DBP) ≥ 90; Normal range = SBP ≤ 139 and DBP ≤ 89; Hypotension = SBP ≤ 90 and DBP ≤ 60 Acronyms: USD = United States dollars, g = grams, Kg Kilograms; m = meters, cm = centimeters, mm = millimeters, Hg = mercury; L = Liters

*The continuous variables are presented as medians with the according 25th and 75th inter quartile range marked

**Table 2:** Infant (one month postpartum) descriptive statistics overall and by maternal anemic status (yes/no) during and one month post-partum

Infant at One Month Postpartum							
Characteristics	Study Population	Maternal Anemic status Pregnancy			Maternal Anemic status One Month Postpartum		
Iron supplementation		(*n* = 56)	(*n* = 28)	0.009	(*n* = 41)	(*n* = 43)	0.415
		Non-Anemic	Anemic	***p*** -value	Non-Anemic	Anemic	***p*** -value
	*N* = 298	(*n* = 229)	(*n* = 65)		(*n* = 164)	(*n* = 134)	
Sex				0.068			0.103
Female	149 (50.0)	121 (52.8)	39 (60.0)		89 (54.3)	60 (44.8)	
Male	149 (50.0)	108 (47.2)	26 (40.0)		75 (45.7)	74 (55.2)	
*Hemoglobin Levels (g/L)					(*n* = 163)	(*n* = 134)	
	121.0 (110.0, 135.0)	130.1 (128.1, 132.1)	99.9 (98.7, 101.2)	0.000	125.3 (122.4, 128.1)	121.1 (117.9, 124.2)	0.054
Anemia				0.000	163	134	0.063
Yes	57 (19.1)	0 (0.0)	56 (86.1)		25 (15.3)	32 (23.9)	
No	240 (80.5)	229 (100.0)	9 (13.9)		138 (85.7)	102 (76.1)	
Missing	1 (0.3)						
*Weight- (g)	4200 (3800, 4500)	4200 (4000, 4300)	4300 (4200, 4500)	0.226	4200 (4100, 4400)	4200 (4100, 4300)	0.668
*Height (cm)	53.0 (51.3, 54.1)	52.5 (52.1, 52.9)	52.9 (52.2, 53.7)	0.306	52.7 (52.2, 53.2)	52.6 (52.1, 53.0)	0.675
*Head circumference (cm)	36.0 (35.1, 37.0)	35.8 (35.5, 36.1)	36.4 (36.1, 36.7)	0.057	36.0 (35.4, 36.6)	36.1 (35.9, 36.3)	0.776
*Mid-upper arm circumference (cm)	11.7 (11.0, 12.3)	11.9 (11.6, 12.2)	11.8 (11.6, 12.0)	0.823	11.9 (11.5, 12.4)	11.8 (11.6, 12.0)	0.621
*Triceps skinfold (cm)	0.8 (0.7, 1.0)	0.8 (0.8, 0.9)	0.9 (0.8, 0.9)	0.387	0.8 (0.8, 0.9)	0.9 (0.8, 0.9)	0.289
*Subscapular skinfold (cm)	0.7 (0.6, 0.9)	0.8 (0.8, 0.9)	0.9 (0.8, 0.9)	0.323	0.8 (0.7, 0.8)	0.8 (0.7, 0.8)	0.378
*Quadriceps skinfold (cm)	1.2 (1.0, 1.4)	1.1 (1.1, 1.2)	1.2 (1.1, 1.3)	0.527	1.2 (1.1, 1.2)	1.1 (1.1, 1.2)	0.291
*Flank skinfold (cm)	0.7 (0.6, 0.9)	0.8 (0.7, 0.8)	0.9 (0.7, 1.2)	0.041	0.8 (0.7, 0.9)	0.8 (0.7, 0.8)	0.408
Doctors visit				0.067			0.266
yes	21 (7.1)	13 (5.7)	8 (12.3)		14 (8.5)	7 (5.2)	
no	277 (92.9)	216 (94.3)	57 (97.7)		150 (91.5)	127 (94.8)	
Iron supplementation		(n=56)	(n=28)	0.009	(n=41)	(n=43)	0.415
Yes	8 (2.7)	2 (3.6)	6 (21.4)		5 (12.2)	3 (7.0)	
No	0 (0.0)	0 (0.0)	0 (0.0)		0 (0.0)	0 (0.0)	
Don’t know	76 (25.5)	54(96.4)	22 (78.6)		36 (87.8)	40 (93.4)	
Missing	214 (71.8)						

The continuous variables are presented as medians with the according 25th and 75th IQR marked with (*). The categorical variables are presented as total numbers and percentages. T-tests are presented as means and 95% confidence intervals (CI). Significance at *p* < 0.05. Acronyms: g = grams; Kg = Kilograms; cm = centimeters; L = Liters; *The continuous variables are presented as medians with the according 25th and 75th inter quartile range marked

### Statistical analysis

The study population was first described using frequencies and percentages for categorical and binary variables and medians with the 25th and 75th IQR for continuous variables. Group differences in maternal anemia were examined using independent t-tests for continuous variables and chi-square tests for categorical variables.

Linear regression models provided Beta estimates and 95% confidence intervals (CI) for the continuous outcomes, including infant anthropometric measurements, as well as maternal postpartum and infant Hb levels. Logistic regression models provided odds ratios (OR) and 95% CI for binary outcomes, such as maternal postpartum and infant anemia (yes/no).

A total of ten models were constructed using maternal anemia during pregnancy as the exposure, and an additional ten models were constructed using maternal Hb levels during pregnancy as the exposure. Furthermore, nine models were estimated for maternal anemia status at one month postpartum, and another nine models for maternal Hb levels at one month postpartum as the exposure variable. Results are presented from both unadjusted (crude) and fully adjusted models. The fully adjusted models controlled for the following covariates: infant biological sex and age, maternal BMI during pregnancy, highest level of educational attainment, marital status, number of antenatal care (ANC) visits, urban versus rural residence, employment status, monthly household income, alcohol consumption, and maternal blood pressure during pregnancy and at one month postpartum.

Data were analyzed using Stata SE 16 [41]. A *p*-value of < 0.05 was used for statistical significance. All participants with complete data on exposures, outcomes, and covariates were included in the analysis. Diagnostic checks confirmed that regression model assumptions—such as normality, homoscedasticity, linearity, and absence of multicollinearity—were met, and no transformations were required.

### Handling of perfect prediction and use of firth logistic regression

The association between maternal anemia during pregnancy and infant anemia at one month postpartum exhibited quasi-complete separation: all anemic infants had anemic mothers. Standard logistic regression failed due to perfect prediction; therefore, we applied Firth’s penalized logistic regression, which addresses separation by introducing a bias-reducing penalty derived from a Jeffreys prior [42]. This method is recommended for small samples or rare outcomes and yields finite, more conservative estimates. Analyses were performed using the firthlogit command in Stata SE 16.

## Results

We identified 298 mothers and their infants at one month postpartum. Table 2 displays the descriptive statistics for the mother during pregnancy and one month postpartum. Mean maternal Hb level during pregnancy was 113 g/L, and 122 g/L at one month post-partum. Most mothers had their first interview during their third trimester of pregnancy (55%, 164/298) followed by the second (36%, n/*N* = 107/298) and then first trimester (9%, 27/298). More mothers were anemic during the postpartum period (45%, 134/295) than during pregnancy (34%, 65/294). The average age of mothers during pregnancy was 27 years, and 90% (269/298) of them were married at that time *(see* Table 2*)*.

Table 1 displays descriptive statistics for the infants at one month postpartum. Infants had a mean weight of 4200 g and a mean height of 53 cm. At one month postpartum, the mean head circumference of infants was 36.0 cm, mean mid-upper arm circumference (MUAC) was 11.7 cm, mean triceps skinfold thickness was 0.8 cm, mean subscapular skinfold thickness was 0.7 cm, mean quadriceps skinfold thickness was 1.2 cm, and mean flank skinfold thickness was 0.7 cm. None of these anthropometric measurements differed significantly between infants of anemic and non-anemic mothers, either during pregnancy or at one month postpartum.

The mean Hb levels of the infants were 121 g/L but differed between infants whose mother was anemic (121 g/L, 95% CI: 117.9, 124.2 g/L) in the postpartum period and infants whose mother was not anemic (125 g/L, 95% CI: 122.4, 128.1 g/L) (*p* = 0.0535). More pronounced differences were observed in relation to maternal anemia during pregnancy. Infants born to mothers who were anemic during pregnancy had substantially lower mean Hb levels (99.9 g/L; 95% CI: 98.7,101.2), compared to infants whose mothers were not anemic during pregnancy (130 g/L; 95% CI: 128.1,132.1) (*p* < 0.001).

Importantly, all 56 infants classified as anemic at one month postpartum were born exclusively to mothers who had anemia during pregnancy. Although this finding suggests a strong association, the perfect prediction observed introduces analytical limitations, and the resulting estimates should be interpreted with care.

Table 3 shows odds ratios and 95% CI, and Beta estimates and 95% CI, for the fully adjusted association between maternal anemia during pregnancy and infant anthropometrics and infant- and maternal postpartum anemic status. In our analysis of the primary outcome, maternal anemia and Hb levels during pregnancy were not associated with infant anthropometric measurements.

**Table 3:** Associations of maternal anemia (yes/no) and hemoglobin levels during pregnancy on infant anemia (yes/no) and infant anthropometrics measurements (one month postpartum)

	Maternal Anemic Status Pregnancy (Hb < 110 g/L)		Maternal Pregnancy Hb levels (g/L)	
	Crude	Fully adjusted	Crude	Fully adjusted
Outcome	OR 95% CI		β-estimates, 95% CI	
	*n* = 294	*n* = 287	*n* = 295	*n* = 288
Infant Anemic Status (Hb < 105 g/L)	7.91*** (5.05, 10.77)	7.36*** (4.69, 10.02)	-	-
Maternal Anemic Status one month postpartum (yes/no) (Hb < 120 g/L)	0.58* (0.03, 1.14)	1.48 (0.81, 2.72)	-	-
	β-estimates, 95% CI			
Infant Hb levels (g/L)	-	-	0.07 (−0.09, 0.23)	0.03 (−0.14, 0.20)
Maternal Hb levels one month postpartum (g/L)	-	-	0.23*** (0.12, 0.34)	0.21*** (0.09, 0.33)
WAZ	0.17 (−0.10, 0.44)	0.20 (−0.09, 0.49)	0.004 (−0.005, 0.01)	0.004 (−0.005, 0.01)
HAZ	0.14 (−0.13, 0.37)	0.08 (−0.21, 0.37)	0.002 (−0.007, 0.01)	0.001 (−0.008, 0.01)
HC for age z scores	0.21* (−0.01, 0.43)	0.21 (−0.02, 0.45)	0.004 (−0.002, 0.01)	0.003 (−0.005, 0.01)
MUAC for age z-scores	−0.03 (−0.30, 0.24)	−0.07 (−0.37, 0.22)	−0.001 (−0.01, 0.008)	−0.0002 (−0.01, 0.009)
Triceps^a^ for age z-scores	0.12 (−0.15, 0.40)	0.13 (−0.16, 0.42)	0.001 (−0.01, 0.01)	−0.0002 (−0.01, 0.01)
Subscapular^a^ for age z-scores	0.14 (−0.14, 0.42)	0.21 (−0.09, 0.51)	0.0001 (−0.01, 0.01)	−0.001 (−0.01, 0.01)
Quadriceps^a^ for age z-scores	0.09 (−0.19, 0.36)	0.21 (−0.07, 0.51)	0.002 (−0.01, 0.01)	0.0002 (−0.01, 0.01)
Flank^a^ for age z-scores	0.05 (−0.08, 0.18)	−0.06 (−0.05, 0.23)	−0.001 (−0.004, 0.003)	−0.001 (−0.01, 0.003)

Estimates are presented as odds ratio (OR) for (logistic regression) and mean differences (β-estimates) for linear regression with the corresponding 95% confidence intervals (CI)

All models are fully adjusted for sex and age of the infant, maternal BMI, highest educational attainment, marital status, antenatal care visits, urban or rural residence, employment status, monthly income, alcohol consumption, and blood pressure

*Significance: *p* < 0.05

** Significance: *p* < 0.01

*** Significance: *p* = 0.000

*HAZ* Height for age z-scores, *WAZ* Weight for age z-scores, *HC* Head circumference, *Hb* Hemoglobin

^a^Skinfold thickness

Regarding our analysis of the secondary outcome, mothers who were anemic during pregnancy are 1.48 times more likely to have anemia one month postpartum (95% CI = 0.81, 2.72; *p* > 0.05), however the estimate was not statistically significant. However, maternal Hb levels during pregnancy and at one month postpartum were positively associated: for an increase of 1 g/L of maternal Hb during pregnancy there was a 0.21 g/L increase of maternal Hb at one month postpartum (β = 0.21, 95% CI = 0.12, 0.34, *p* < 0.01).

Infants born to mothers who were anemic during pregnancy were 7.36 times more likely to have anemia compared to infants whose mothers were not anemic during pregnancy (95% CI = 4.69, 10.02, *p* < 0.01). These estimates were obtained using Firth’s penalized logistic regression, which corrects for small-sample bias and separation. Given this modeling approach, the reported estimates should be interpreted with caution, as they may not reflect unbiased population-level associations. Notably, maternal hemoglobin levels during pregnancy were not significantly associated with infant hemoglobin levels at one month postpartum (β = 0.03; 95% CI: − 0.14, 0.20). For comparison, we provide results based on the previous WHO 2011 anemia classification criteria in the Supplementary Information, which did not result in perfect prediction and yielded more conservative estimates.

Table 4 presents the results for the fully adjusted associations between maternal postpartum anemia with infant- anemia and anthropometric for age z scores and the results for the association of maternal postpartum Hb levels with infant Hb levels and anthropometric for age- z-scores. Regarding our primary outcome, no statistically significant associations were observed between maternal anemia and Hb levels and anemic status at one month-postpartum and infant anthropometric measurements.

**Table 4:** Associations of maternal postpartum anemia (yes/no) and hemoglobin levels on infant anemia (yes/no) and infant anthropometrics for age z-scores at one month postpartum

	Maternal Anemic Status Postpartum (Hb < 120 g/L)		Maternal Postpartum HB levels (g/L)	
	Crude	Fully adjusted	Crude	Fully adjusted
Outcome	OR 95% CI		β-estimates, 95% CI	
	*n* = 297	*n* = 287	*n* = 297	*n* = 287
Infant Anemic Status (Hb < 110 g/L)	0.55 (−0.03, 1.13)	1.45 (0.76, 2.76)	-	-
	β-estimates, 95% CI			
	*n* = 298	*n* = 288	*n* = 298	*n* = 288
Infant Hb levels (g/L)	-	-	0.30** (0.13, 0.47)	0.23** (0.05, 0.40)
WAZ	−0.05 (−0.28, 0.18)	−0.09 (−0.34, 0.15)	−0.004 (−0.01, −0.005)	−0.003 (−0.01, 0.01)
HAZ	−0.05 (−0.28, 0.18)	−0.03 (−0.30, 0.21)	0.0004 (−0.01, 0.01)	0.001 (−0.01, 0.01)
HC for age z-scores	0.03 (−0.19, 0.26)	0.08 (−0.09, 0.29)	−0.006 (−0.01, 0.003)	−0.007 (−0.02, 0.0002)
MUAC for age z-scores	−0.06 (−0.29, 0.17)	−0.08 (−0.33, 0.16)	0.005 (−0.003, 0.01)	0.005 (−0.004, 0.01)
Triceps^a^ for age z-scores	0.12 (−0.11, 0.35)	0.13 (−0.12, 0.37)	−0.004 (−0.01, 0.01)	−0.004 (−0.01, 0.01)
Subscapular^a^ for age z-scores	−0.10 (−0.33, 0.13)	−0.05 (−0.30, 0.20)	0.002 (−0.01, 0.01)	0.001 (−0.01, 0.01)
Quadriceps^a^ for age z-scores	−0.12 (−0.35, 0.11)	−0.04 (−0.28, 0.20)	0.003 (−0.01, 0.01)	−0.001 (−0.01, 0.01)
Flank^a^ for age z-scores	−0.003 (−0.11, 0.10)	−0.11 (−0.10, 0.13)	−0.0003 (−0.005, 0.004)	0.001 (−0.01, 0.004)

Estimates are presented as odds ratio (OR) for (logistic regression) and mean differences (β-estimates) for linear regression with the corresponding 95% confidence intervals (CI)

All models are fully adjusted for sex and age of the infant, maternal BMI, highest educational attainment, marital status, antenatal care visits, urban or rural residence, employment status, monthly income, alcohol consumption, and blood pressure

*Significance: *p* < 0.05

** Significance: *p* < 0.01

*HAZ* Height for age z-scores, *WAZ* Weight for age z-scores, *HC* Head circumference, *Hb* Hemoglobin

^a^ Skinfold thickness

Regarding our secondary outcome, there was a 0.2 increase in the infant’s Hb level for every one unit increase in the mother’s Hb level at one month postpartum (β = 0.23, 95% CI: 0.05, 0.40, *p* < 0.01). No further associations reached significance.

There was no association between maternal anemia status at one month postpartum and infant anemic status. However, the trend shows that that infants whose mothers were anemic at one month postpartum were 1.45 times more likely to be anemic themselves (95% CI = 0.76, 2.76, *p* > 0.05), however the estimate was not statistically significant.

## Discussion

The primary objective of this study was to examine the potential impact of maternal anemia during pregnancy and at one month postpartum on the anthropometric measurements of healthy, term infants at one month postpartum. At one month postpartum, infant anthropometric measurements—including head circumference, MUAC, and various skinfold thicknesses—were within expected ranges for healthy, term infants according to the WHO growth standards [31] and showed no significant variation by maternal anemia status during pregnancy. To our knowledge this is the first study investigating the relationship between maternal anemia and healthy weight in term infants. A possible explanation that we could not find an association might be because the relationship between anemia and infant anthropometric measurements depends on the degree of severity of anemia [4, 5]. This relationship is demonstrated by the results of a study conducted in 2009 by Al-Mendalawi et al. in Istanbul, Turkey. They showed that mean height, weight, head-, and chest -circumference of neonates in the severe anemic group was lower than in the mild anemic group [5]. Due to the lack of variability in the severity of anemia, our study was not able to investigate the impact of the severity of anemia on infant anthropometric measurements. Moreover, the infants in our study were only one month old, a period when infant growth, particularly weight, is still stabilizing. It is well established that most infants regain their birth weight only by the end of the first month of life [31] and have not yet demonstrated substantial additional growth. This early timing of measurement may therefore have limited our ability to detect more subtle differences in anthropometric outcomes.

Our secondary objective was to investigate the association between maternal anemia during pregnancy and anemia outcomes in both mothers and infants at one month postpartum. Under the updated 2024 WHO hemoglobin thresholds, this association exhibited quasi-complete separation: all anemic infants were born to anemic mothers. To address this, we applied Firth’s penalized logistic regression, which adjusts for small-sample bias and separation. The resulting odds ratio of 7.4 suggests a substantial increase in the odds of infant anemia among infants of anemic mothers, underscoring the intergenerational dimension of nutritional vulnerability [43]. Although Firth regression yields conservative estimates, the association remains substantively meaningful. For transparency and comparability with previous studies, we present the corresponding analysis using the 2011 WHO anemia thresholds in the supplementary information. Additionally, the results show a 0.2 increase in Hb levels for infants at one month, which is significantly associated with maternal postpartum Hb levels. This finding reinforces the broader link between maternal health and early-life outcomes [44]. Our findings align with existing evidence that maternal anemia contributes to both maternal and neonatal adverse health risks [44–46].

Moreover, our results reflect existing evidence suggesting that exclusively breastfed infants born to anemic mothers have lower Hb levels compared to infants born to non-anemic mothers [44, 47]. However, since this study assessed hemoglobin concentrations only at the one month postpartum timepoint and did not explore longitudinal trends, caution is warranted in interpreting Hb levels as a predictor for anemia [14, 48]. For example, a systematic review by Sanni O.B. et al., 2020 stated that correlations of hematological indices between mother and neonate, such as Hb levels, are negligible, due to the lack of further information regarding the source of the cause of anemia [49]. Thus, the true association between maternal postpartum anemia on infant anemia at one month post-partum is still unclear. However, we hypothesize that it is an indirect association, indirect through the association of postpartum anemia with postpartum depression, fatigue [12], impaired lactation, and early cessation of breastfeeding [11] and impaired mother and child bonding [12] which may lead to poor care and nutrition of the infant leading to impaired growth and health of the infant. Our results highlight the need to prevent maternal anemia during pregnancy and throughout the postpartum period. Further studies should investigate the association between maternal postpartum health, such as anemia, and infant growth to validate the findings of the present study.

Our study population consisted of exclusively breastfed healthy term singleton infants and results show that infants were on average non-anemic with a mean hemoglobin level of 121 g/L (CI = 108, 135 g/L). However, it is unclear if breast milk alone (i.e. exclusive breastfeeding) is sufficient for providing optimum iron and supporting child growth, especially among mothers with anemia [21]. On one hand, our results may suggest a protective function of breastmilk against anemia, considering that exclusive breastfeeding is considered to be the ideal nutrition for infants and sufficiently supports optimal growth for the first six months of life [19, 20]. On the other hand, our results could also support evidence that there is no relationship between maternal anemia and iron concentration in breast milk [50]. Zavaleta et al. measured iron and lactoferrin concentrations in milk after two and 30 days postpartum [50]. For anemic mothers, iron concentrations in milk were 0.90 and 0.38 mg/L at 2- and 30-days post-partum, and for non-anemic mothers, were 0.80 and 0.35 mg/ml, respectively. They concluded that milk iron values were within the range previously reported for healthy mothers, and therefore, that maternal anemia did not affect milk iron or lactoferrin concentrations [50]. However, other studies have reported that among anemic mothers, infants who were exclusively breastfed may become iron deficient [19] — leading to reports that exclusively breastfed infants should receive iron supplementation from 4 months of age [20] and/or complementary foods be introduced before 6 months of age [11, 22]. These studies underline that the link between the iron status of exclusively breastfed infants in relation to iron levels of breast milk and maternal anemia is still not completely understood and needs further investigation.

### Limitations & strengths of the study

There are several limitations to this study. We were not able to investigate dietary iron bioavailability (iron compounds, heme, and non-heme), which fails to account for other causes of anemia, for example nutritional deficiencies (iron and vitamin A), infectious disorders (malaria, HIV, tuberculosis), and hemoglobinopathies. Knowledge of the dietary iron intake of the mother and additional biomarkers, such as serum ferritin, are necessary to better define the true cause of anemia. Data on demographic characteristics are self-reported by the participants, which could possibly lead to social desirability and information bias. A further limitation is the lack of data on iron supplementation in newborns for most participants, which may have influenced infant hematologic outcomes and limited control for this potential confounder. However, iron supplementation or routine anemia screening in infants is not standard practice in Lao PDR, making supplementation among healthy infants unlikely.

Another key limitation of our study is the application of WHO growth standards for skinfold thickness and MUAC starting at one month of age, despite official recommendations beginning at 3 months, due to the absence of normative data for younger infants. Similarly, in the absence of WHO hemoglobin thresholds for infants under six months, we applied the six months cutoff to one month old infants. These approaches, while aligned with current standards, may introduce some imprecision or misclassification due to age-related physiological differences.

Due to the study design, investigating exclusively breastfed infants, we cannot rule out selection bias. There is possible residual confounding from other possible factors influencing anemia and anthropometric outcomes. For example, we were not able to control for diet; infectious diseases, hemoglobinopathies severity of anemia and postpartum depression, and the resulting mother and child interaction. However, our study is focused on the healthy term, singleton, and exclusively breastfed infants which helps remove additional biases.

Finally, it is important to interpret our findings considering the specific study population and timing of data collection. This analysis focused on healthy, term infants (≥ 37 weeks of gestation) from Southeast Asia who were not classified as low birth weight. All anthropometric measurements were taken at one month postpartum, a time when early growth may not yet reflect longer-term nutritional or developmental outcomes. Therefore, our findings are most relevant to early growth in healthy, full-term infants and may not apply to preterm, low birth weight and formula fed populations. Due to quasi-complete separation in the exposure-outcome relationship, we used Firth’s penalized logistic regression. While this approach reduces bias, the resulting estimates reflect penalized likelihoods and should be interpreted with caution.

Despite these limitations, the longitudinal nature of the study strengthens the argument that there is a causal relationship between maternal anemia during pregnancy and maternal anemia and infant anemia, at one month postpartum. While the study population is unique and context-specific to Southeast Asia, the analytical framework and methodological approach are applicable to other settings with similar environmental, nutritional, and socio-political characteristics. Moreover, this study contributes to the limited research and knowledge on the impact of maternal anemia on infant anemic status and anthropometric measurements.

## Conclusions

Our results provide evidence that maternal anemia during pregnancy and at one month postpartum plays an important role on infant health, which may be impaired if maternal and infant anemia postpartum remains undiagnosed and untreated. This underscores maternal nutrition as a key determinant of infant growth and well-being. However, further research is needed to better understand the mechanisms through which postpartum maternal anemia influences infant health and growth outcomes. Additionally, validated growth standards for infants younger than 3 months are urgently needed to improve early nutritional assessment.

Our study and results contribute to the WHO statement that the postpartum period is a critical and often forgotten phase in the lives of women and newborns [51]. From a public health perspective, this study supports the development and implementation of policies that strengthen continuity of care across the perinatal period, with particular attention to postpartum maternal anemia and nutritional support. We further highlight the necessity of including both pregnancy and postpartum timepoints when investigating the impact of maternal anemia on maternal health and infant health, growth, and development.

## Supplementary Information

Supplementary material 1.

## Data Availability

Data described in the manuscript, code book, and analytic code will be made available upon reasonable request pending email to the corresponding author.

## Abbreviations

CI: Confidence interval
cm: Centimeters
g: Grams
HAZ: Height for age z-scores
Hb: Hemoglobin
HC: Head circumference
IDA: Iron deficiency anemia
ID: Iron deficiency
Kg: Kilogram
L: Liter
Lao PDR: Lao People's Democratic Republic
m: Meters
mm: Millimeters
MUAC: Mid-upper arm circumference
OR: Odds ratio
PTB: Preterm birth
RBC: Red blood cells
SD: Standard deviation
STEB: Social transfer for exclusive breastfeeding
USD: US Dollars
VITERBI: Vientiane multi generational birth cohort
WAZ: Weight for age z-scores
WHO: World health organization
