# Supplementary material for: Maternal and infant outcomes of pregnancy anemia at one month postpartum: a cohort analysis
Source: BMC Pregnancy Childbirth. 2025 Oct 8;25:1039. doi: 10.1186/s12884-025-08137-3 (PMC12505624; doi:10.1186/s12884-025-08137-3)
Supplement: Supplementary file 1 — Supplementary material 1. [file 12884_2025_8137_MOESM1_ESM.pdf]

Supplementary Information

Table 1: Maternal descriptive statistics overall and by maternal anemic status (yes/no) during (a) pregnancy and at (b) one-month post-partum. Anemia thresholds were determined according to the World Health Organization (WHO) guidelines established in 2011.

(a) Pregnancy

**Characteristics**	**Study Population**	**Maternal Anemic status Pregnancy**			**Maternal Anemic status One-Month Postpartum**		
		**Non-Anemic**	**Anemic**	***p*-value**	**Non-Anemic**	**Anemic**	***p*-value**
	N= 298	(n=195)	(n=100)		(n=161)	(n=134)	
**Area**				0.036*			0.86
Urban	154 (51.7)	109 (55.9)	43 (43.0)	-	84 (51.2)	70 (52.2)	-
Rural	144 (48.3)	86 (44.1)	57 (57.0)	-	80 (48.8)	64 (47.8)	-
***Age** (years) (mean, CI)	28 (24, 31)	27 (26, 28)	27 (26 29)	0.5986	28 (27, 29)	26 (25, 28)	0.0673
***Hemoglobin** (g/L) (mean, CI)	113.0 (105.0, 122.0)	-	-	-	116.3 (114.4, 118.2)	110.3 (108.0, 112.7)	0.0001**
**Anemia**							0.018*
Yes	100 (33.0)	-	-	-	79 (40.5)	55 (55.0)	-

Characteristics	Study Population	Maternal Anemic status Pregnancy			Maternal Anemic status One-Month Postpartum		
		Non-Anemic	Anemic	*p*-value	Non-Anemic	Anemic	*p*-value
No	195 (64.4)	-	-	-	116 (59.5)	45 (45.0)	-
***Weight** (Kg)	56.9 (50.6, 64.1)	60.1 (58,4, 61.8)	55.8 (54.1, 57.5)	0.0021**	58.5 (56.8, 60.3)	58.8 (56.9, 60.8)	0.8131
***Height** (m)	1.5 (1.5, 1.6)	1.5 (1.5, 1.6)	1.5 (1.5, 1.5)	0.4207	1.5 (1.5, 1.6)	1.5 (1.5, 1.56)	0.5240
**BMI** (Kg/m^^{2})				0.177	N=(161)	N=134	0.374
Underweight	7 (2.3)	4 (2.1)	3 (3.0)	-	5 (3.1)	2 (1.5)	-
Normal	169 (56.7)	105 (53.9)	64 (64.0)	-	96 (59.6)	73 (54.5)	-
Overweight	119 (39.9)	86 (44.1)	33 (33.0)	-	60 (37.3)	59 (44.0)	-
**Blood Pressure** (mm Hg)		(n=193)	(n=99)	0.737			
Hypertension	-	-	-				
Normal range	260 (87.3)	171 (88.6)	89 (89.9)				
Hypotension	32 (10.7)	22 (11.4)	10 (10.1)				
***Income** /per month (USD)	87.0 (17.4, 116.0)	139.9 (95.8, 183.9)	193.6 (72.3, 314.8)	0.3177	145.01 (95.5, 194.5)	173.75 (80.4, 267.1)	0.5741

Characteristics	Study Population	Maternal Anemic status Pregnancy			Maternal Anemic status One-Month Postpartum		
		Non-Anemic	Anemic	*p*-value	Non-Anemic	Anemic	*p*-value
**Work Status**				0.401	161	134	0.134
Unemployed	98 (31.5)	62 (31.8)	32 (32.0)	-	46 (28.6)	48 (35.8)	-
Employed	131 (44.0)	91 (46.7)	40 (40.0)	-	80 (49.7)	51 (38.1)	-
Other	70 (23.5)	42 (21.5)	28 (28.0)	-	35 (21.7)	35 (26.1)	-
**Education**				0.785			0.320
Primary	76 (25.5)	52 (26.7)	24 (24.0)	-	37 (23.0)	39 (29.1)	-
Secondary	119 (39.9)	76 (39.0)	43 (43.0)	-	64 (39.8)	55 (41.0)	-
University/ Higher	100 (33.6)	67 (34.3)	33 (33.0)	-	60 (37.3)	40 (29.9)	-
**Marital Status**				0.936			0.738
Never married	26 (8.7)	17 (8.7)	9 (9.0)	-	15 (9.3)	11 (8.2)	-
Currently married	269 (90.3)	178 (91.3)	91 (91.0)	-	146 (90.7)	123 (91.8)	-
**Alcohol con-sumption (yes/no)**				0.271			0.529
Yes	62 (20.8)	36 (18.5)	26 (26.0)		18 (11.2)	12 (9.0)	
No	233 (78.2)	159 (81.5)	74 (74.0)		143 (88.8)	122 (91.0)	

Characteristics	Study Population	Maternal Anemic status Pregnancy			Maternal Anemic status One-Month Postpartum		
		Non-Anemic	Anemic	*p*-value	Non-Anemic	Anemic	*p*-value
**Antenatal care visits**	3.0 (2.0, 5.0)	3.6 (2.60, 4.6)	3.2 (2.83, 3.5)	0.5317	3.6 (2.46, 4.8)	3.2 (2.90, 3.5)	0.4981
**Iron Supple-mentation**				0.377			0.529
Yes	265 (88.9)	173 (88.7)	92 (92.0)	-	143 (88.8)	122 (91.0)	-
No	30 (10.1)	22 (11.3)	8 (8.0)	-	18 (11.2)	12 (9.0)	-

(b) One-month post-partum

Characteristics	Study Population	Maternal Anemic status Pregnancy			Maternal Anemic status One-month Postpartum		
		Non-Anemic	Anemic	*p*-value	Non-Anemic	Anemic	*p*-value
	N= 298	(n=195)	(n=100)		(n=147)	(n=128)	
***Hemoglobin level** (g/L)	122.0 (113.0, 131.0)	122.7 (120.9, 124.6)	119.2 (116.9, 121.4)	0.0213*	-	-	-
**Anemia**				0.018*	-	-	-
Yes	134 (45.0)	79 (40.5)	55 (55.00)	-	-	-	-
No	164 (55.0)	116 (59.5)	45 (45.00)	-	-	-	-

Characteristics	Study Population	Maternal Anemic status Pregnancy			Maternal Anemic status One-Month Postpartum		
		Non-Anemic	Anemic	*p*-value	Non-Anemic	Anemic	*p*-value
**Blood Pressure** (mm Hg)		-	-	-	-	-	0.748
Hypertension	9 (3.0)	-	-	-	6 (3.7)	3 (2.2)	-
Normal range	281 (94.3)	-	-	-	154 (93.9)	127 (94.8)	-
Hypotension	8 (2.7)	-	-	-	4 (2.4)	4 (3.0)	-

The continuous variables are presented as medians with the according 25th and 75th inter quartile range marked (*). The categorical variables are presented total numbers and percentages. T-tests are presented as means and 95% confidence intervals (CI). Significance at $p<0.05$. BMI is categorized as follows: Underweight (>18.5); Normal weight (18.5 - <25) and Overweight (25.0 to 30+). Blood Pressure is categorized as follows: Hypertension = systolic blood pressure (SBP) ≥140 and diastolic blood pressure (DBP) ≥ 90; Normal range = SBP ≤ 139 and DBP ≤ 89; Hypotension= SBP ≤ 90 and DBP ≤ 60. Acronyms: USD=United States dollar; g = gram, Kg = Kilogram; m= meter, cm= centimeter; mm= millimeter; Hg= mercury; L= Liter

Table 2: Infant (one-month postpartum) descriptive statistics overall and by maternal anemic status (yes/no) during and after pregnancy (one-month post-partum. Anemia thresholds were determined according to the World Health Organization (WHO) guidelines established in 2011.

Infant

**Characteristics**	**Study Population**	**Maternal Anemic status Pregnancy**			**Maternal Anemic status One-month Postpartum**		
		**Non-Anemic**	**Anemic**	***p*-value**	**Non-Anemic**	**Anemic**	***p*-value**
	N= 298	(n=195)	(n=100)		(n=164)	(n=134)	
**Sex**				0.346			0.103
Female	149 (50.0)	94 (48.2)	54 (54.0)	-	89 (54.3)	60 (44.8)	-
Male	149 (50.0)	101 (51.8)	46 (46.0)	-	75 (45.7)	74 (55.2)	-
***Hemoglobin** Levels (g/L)		(n=195)	(n=99)		(n=163)	(n=134)	
	121.0 (110.0, 135.0)	124.07 (121.49, 126.66)	122 (118.32, 126.00)	0.4054	125.26 (122.39, 128.14)	121.07 (117.91, 124.24)	0.0535*
**Anemia**							
Yes	74 (24.8)						
No	223 (74.8)						
***Weight- (kg)**	4.2 (3.80, 4.5)	4.27 (4.11, 4.44)	4.11 (4.01, 4.21)	0.1779	4.25 (4.06, 4.44)	4.19 (4.11, 4.29)	0.6679
***Height (cm)**	53.0 (51.3, 54.1)	52.54 (52.10, 52.98)	52.78 (52.27, 53.28)	0.5177	52.70 (52247, 53.17)	52.56 (52.07, 53.04)	0.6748

Infant

Characteristics	Study Population	Maternal Anemic status Pregnancy			Maternal Anemic status One-month Postpartum		
		Non-Anemic	Anemic	*p*-value	Non-Anemic	Anemic	*p*-value
***Head circumference** (cm)	36.0 (35.1, 37.0)	36.0 (35.6, 6.3)	35.8 (35.5, 36.2)	0.5414	36.0 (35.4, 36.6)	36.1 (35.9, 36.3)	0.7758
***Mid-upper arm circumference** (cm)	11.7 (11.0, 12.3)	11.9 (11.53, 12.3)	11.8 (11.56, 12.1)	0.7388	11.93 (11.5, 12.4)	11.80 (11.6, 12.0)	0.6205
***Triceps skinfold** (cm)	0.8 (0.7, 1.0)	0.8 (0.8, 0.8)	0.9 (0.8, 0.9)	0.8698	0.8 (0.8, 0.9)	0.9 (0.8, 0.9)	0.2890
***Subscapular skinfold** (cm)	0.7 (0.6, 0.9)	0.8 (0.8, 0.8)	0.8 (0.7, 0.8)	0.3392	0.8 (0.8, 0.8)	0.8 (0.7, 0.8)	0.3781
***Quadriceps skinfold** (cm)	1.2 (1.0, 1.4)	1.2 (1.1, 1.2)	1.2 (1.1, 1.2)	0.6444	1.2 (1.1, 1.2)	1.2 (1.1, 1.2)	0.2909
***Flank skinfold** (cm)	0.7 (0.6, 0.9)	0.8 (0.7, 0.9)	0.8 (0.7, 0.8)	0.7605	0.8 (0.7, 0.9)	0.8 (0.7, 0.9)	0.4080
**Doctors visit**				0.168			0.266
yes	21 (7.1)	11 (5.6)	10 (10.0)	-	14 (8.5)	7 (5.2)	-
no	277 (93.0)	184 (94.4)	90 (90.0)	-	150 (91.5)	127 (94.8)	-

Infant

**Characteristics**	**Study Population**	**Maternal Anemic status Pregnancy**			**Maternal Anemic status One-month Postpartum**		
		**Non-Anemic**	**Anemic**	***p*-value**	**Non-Anemic**	**Anemic**	***p*-value**
**Iron supplementation**		(n=51)	(n=33)	0.497	(n=41)	(n=43)	0.415
Yes	8 (2.7)	4(7.8)	4 (12.1)	-	5 (12.2)	3 (7.0)	-
No	0 (0.0)	-	-	-	0 (0.0)	0 (0.0)	-
Don't know	76 (25.5)	47(92.2)	29 (87.9)	-	36 (87.8)	40 (93.4)	-

The continuous variables are presented as medians with the according 25th and 75th IQR marked with (*). The categorical variables are presented as total numbers and percentages. T-tests are presented as means and 95% confidence intervals (CI). Significance at $p<0.05$. Acronyms: g = gram; Kg = Kilogram; cm= centimeter; L= Liter

Table 2: Associations of pregnancy anemia (yes/no) and pregnancy hemoglobin levels on infant anemia (yes/no) and infant anthropometrics measurements. Anemia thresholds were determined according to the World Health Organization (WHO) guidelines established in 2011.

	**Maternal Anemia Pregnancy Hb < 110 g/L**		**Maternal Anemia Pregnancy Hemoglobin levels (g/L)**	
	Crude	Fully adjusted	Crude	Fully adjusted
**Outcome**	OR 95% CI		β-estimates, 95% CI	
	n=298	n=292	n=298	n=291
**Infant Anemia Hb < 110 g/L**	0.35 (-0.19, 0.89)	1.52 (0.84, 2.74)	0.07 (-0.09, 0.23)	0.04 (-0.12, 0.21)
**Maternal Anemia Postpartum (yes/no)**	0.58** (0.09, 1.07)	2.06** (1.21, 3.48)	0.23** (0.12, 0.34)	0.24** (0.13, 0.34)
	β-estimates, 95% CI		β-estimates, 95% CI	
**WAZ**	-0.16 (-0.41, 0.08)	-0.09 (-0.34, 0.16)	0.004 (-0.005, 0.01)	0.002 (-0.007, 0.01)
**HAZ**	0.07 (-0.16, 0.32)	0.12 (-0.13, 0.37)	0.002 (-0.007, 0.01)	0.002 (-0.007, 0.01)
**HC for age z scores**	-0.06 (-0.25, 0.13)	-0.02 (-0.22, 0.18)	0.004 (-0.002, 0.01)	0.004 (-0.004, 0.01)
**MUAC for age z-scores**	-0.04 (-0.28, 0.20)	-0.06 (-0.31, 0.19)	-0.001 (-0.009, 0.008)	-0.001 (-0.009, 0.008)
**Triceps[a] for age z-scores**	0.02 (-0.22, 0.26)	0.06 (-0.19, 0.32)	0.001 (-0.007, 0.009)	-0.001 (-0.01, 0.01)
**Subscapular[a] for age z-scores**	-0.12 (-0.36, 0.12)	-0.08 (-0.33, 0.17)	0.0001 (-0.01, 0.01)	-0.001 (-0.01, 0.01)
**Quadriceps[a] for age z-scores**	-0.06 (-0.30, 0.19)	-0.06 (-0.32, 0.19)	0.002 (-0.01, 0.01)	0.001 (-0.01, 0.01)
**Flank[a] for age z-scores**	-0.04 (-0.28, 0.20)	-0.06 (-0.32, 0.19)	-0.001 (-0.01, 0.01)	-0.0003 (-0.01, 0.01)

*Significance: p < 0.05

** Significance: p < 0.01

[a] Skinfold thickness

Estimates are presented as odds ratio (OR) for (logistic regression) and mean differences (β-estimates) for linear regression with the corresponding 95% confidence intervals (CI).

All models are fully adjusted for sex and age of the infant, maternal BMI, highest educational attainment, marital status, antenatal care visits, urban or rural residence, employment status, monthly income, alcohol consumption, and blood pressure.

HAZ= Height for age z-scores, WAZ= Weight for age z-scores, HC= Head circumference

Table 4: Associations of postpartum anemia (yes/no) and hemoglobin levels on infant anemia (yes/no) and infant anthropometrics for age z-scores. Anemia thresholds were determined according to the World Health Organization (WHO) guidelines established in 2011.

	**Maternal Anemia Postpartum Hb < 120 g/L**		**Maternal Anemia Postpartum Hemoglobin levels (g/L)**	
	Crude	Fully adjusted	Crude	Fully adjusted
**Outcome**	OR 95% CI		β-estimates, 95% CI	
	n=298	n=292	n=298	n=292
**Infant Anemia Hb < 110 g/L**	0.55* (0.02, 1.08)	1.54 (0.88, 2.71)	0.30** (0.13, 0.47)	0.23** (0.06, 0.40)
	β-estimates, 95% CI			
**WAZ**	-0.05 (-0.28, 0.18)	-0.08 (-0.31, 0.15)	-0.004 (-0.01, -0.005)	-0.003 (-0.01, 0.01)
**HAZ**	-0.05 (-0.28, 0.18)	-0.06 (-0.30, 0.17)	0.0004 (-0.01, 0.01)	0.001 (-0.01, 0.01)
**HC for age z-scores**	0.03 (-0.19, 0.26)	0.09 (-0.09, 0.29)	-0.006 (-0.01, -0.003)	-0.007 (-0.01, 0.001)
**MUAC for age z-scores**	-0.05 (-0.29, 0.17)	-0.09 (-0.33, 0.14)	0.005 (-0.003, 0.01)	0.005 (-0.004, 0.01)
**Triceps[a] for age z-scores**	0.12 (-0.11, 0.35)	0.14 (-0.10, 0.38)	-0.004 (-0.01, 0.01)	-0.004 (-0.01, 0.004)
**Subscapular[a] for age z-scores**	-0.10 (-0.33, 0.13)	-0.09 (-0.34, 0.15)	0.002 (-0.01, 0.01)	0.002 (-0.01, 0.02)
**Quadriceps[a] for age z-scores**	-0.12 (-0.35, 0.11)	-0.05 (-0.29, 0.19)	0.003 (-0.01, 0.01)	0.0003 (-0.01, 0.01)
**Flank[a] for age z-scores**	-0.09 (-0.32, 0.13)	-0.10 (-0.35, 0.14)	0.0004 (-0.01, 0.01)	0.001 (-0.01, 0.01)

*Significance: $p < 0.05$

** Significance: $p < 0.01$

[a] Skinfold thickness

Estimates are presented as odds ratio (OR) for (logistic regression) and mean differences (β-estimates) for linear regression with the corresponding 95% confidence intervals (CI).

All models are fully adjusted for sex and age of the infant, maternal BMI, highest educational attainment, marital status, antenatal care visits, urban or rural residence, employment status, monthly income, alcohol consumption, and blood pressure.

HAZ= Height for age z-scores, WAZ= Weight for age z-scores, HC= Head circumference

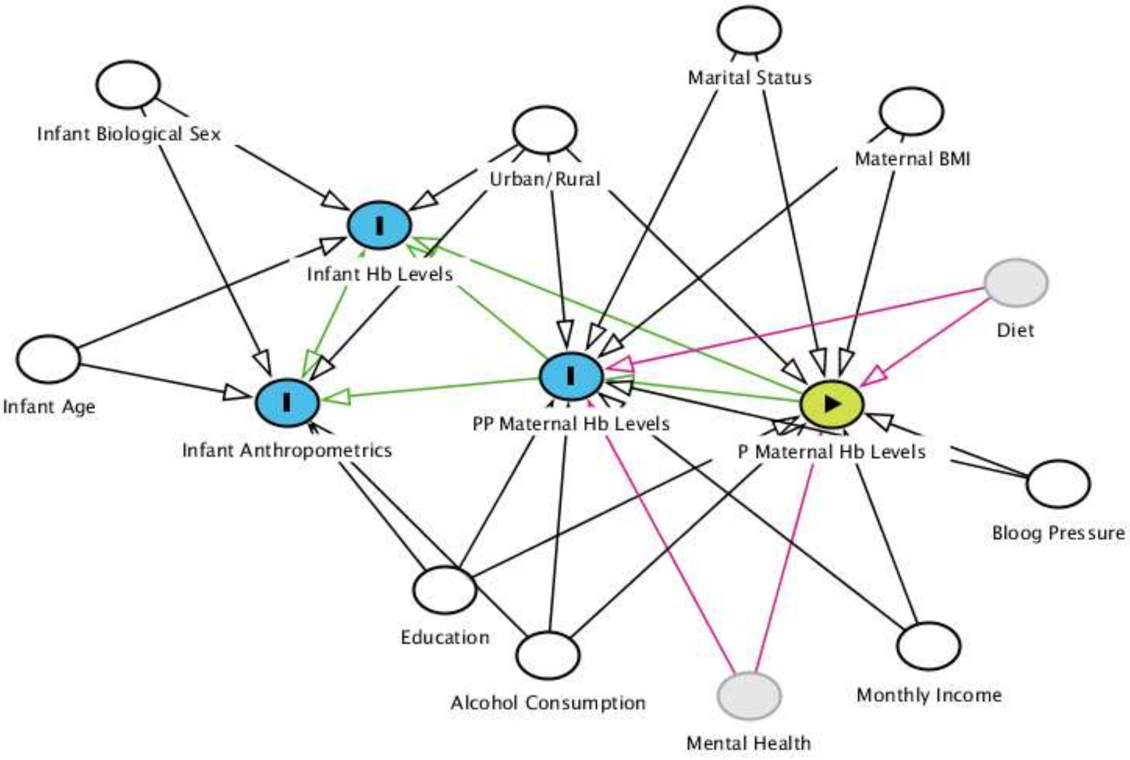

Figure 1: Directed Acyclic Graph (DAG) to inform confounder selection. P= Pregnancy; PP= Post-partum; Hb= Hemoglobin; BMI = Body Mass Index
